# Sedative Application

**Published:** 1859-04

**Authors:** 


*Sedative Application.*—Extract of belladonia, one drachm and a half; liquify with from thirty to forty-five drops of laudanum; triturate in a mortar, and add one drachm of chloroform. Spread this three or four times a day on the region affected with neuralgia or acute inflammation. It will adhere to the skin longer than an ointment.—Dr. Diday.

### *Treatment of Itch.*

M. Beau considers the essence of turpentine well rubbed into the body as the most effectual and rapid means of destroying the acari and their ova. For those patients who dislike its smell, or in whom it excites cutaneous irritation, he employs Helmerich's ointment, (lard 8, sulphur 2 parts, and subc. potass 1 part,) or Bourguignons—powdered staphisagria 300, and lard 500 parts. Before putting them on again the clothes must be effectually purified or the disease may be reproduced, even at the end of many days. This is done effectually by exposing them to a temperature of from 80 deg. to 100 deg. R.—*Gazette des Hôpitaux*, 1858, No. 37.—*London Lancet.*

